# Multiple Phenotypic Changes Define Neutrophil Priming

**DOI:** 10.3389/fcimb.2017.00217

**Published:** 2017-05-29

**Authors:** Irina Miralda, Silvia M. Uriarte, Kenneth R. McLeish

**Affiliations:** ^1^Department of Microbiology, University of Louisville School of MedicineLouisville, KY, United States; ^2^Department of Medicine, University of Louisville School of MedicineLouisville, KY, United States; ^3^Robley Rex VA Medical CenterLouisville, KY, United States

**Keywords:** neutrophils, priming, cytokines, chemotaxis, apoptosis, phagocytosis, respiratory burst, exocytosis

## Abstract

Exposure to pro-inflammatory cytokines, chemokines, mitochondrial contents, and bacterial and viral products induces neutrophils to transition from a basal state into a primed one, which is currently defined as an enhanced response to activating stimuli. Although, typically associated with enhanced generation of reactive oxygen species (ROS) by the NADPH oxidase, primed neutrophils show enhanced responsiveness of exocytosis, NET formation, and chemotaxis. Phenotypic changes associated with priming also include activation of a subset of functions, including adhesion, transcription, metabolism, and rate of apoptosis. This review summarizes the breadth of phenotypic changes associated with priming and reviews current knowledge of the molecular mechanisms behind those changes. We conclude that the current definition of priming is too restrictive. Priming represents a combination of enhanced responsiveness and activated functions that regulate both adaptive and innate immune responses.

## Introduction

**Figure 1:**
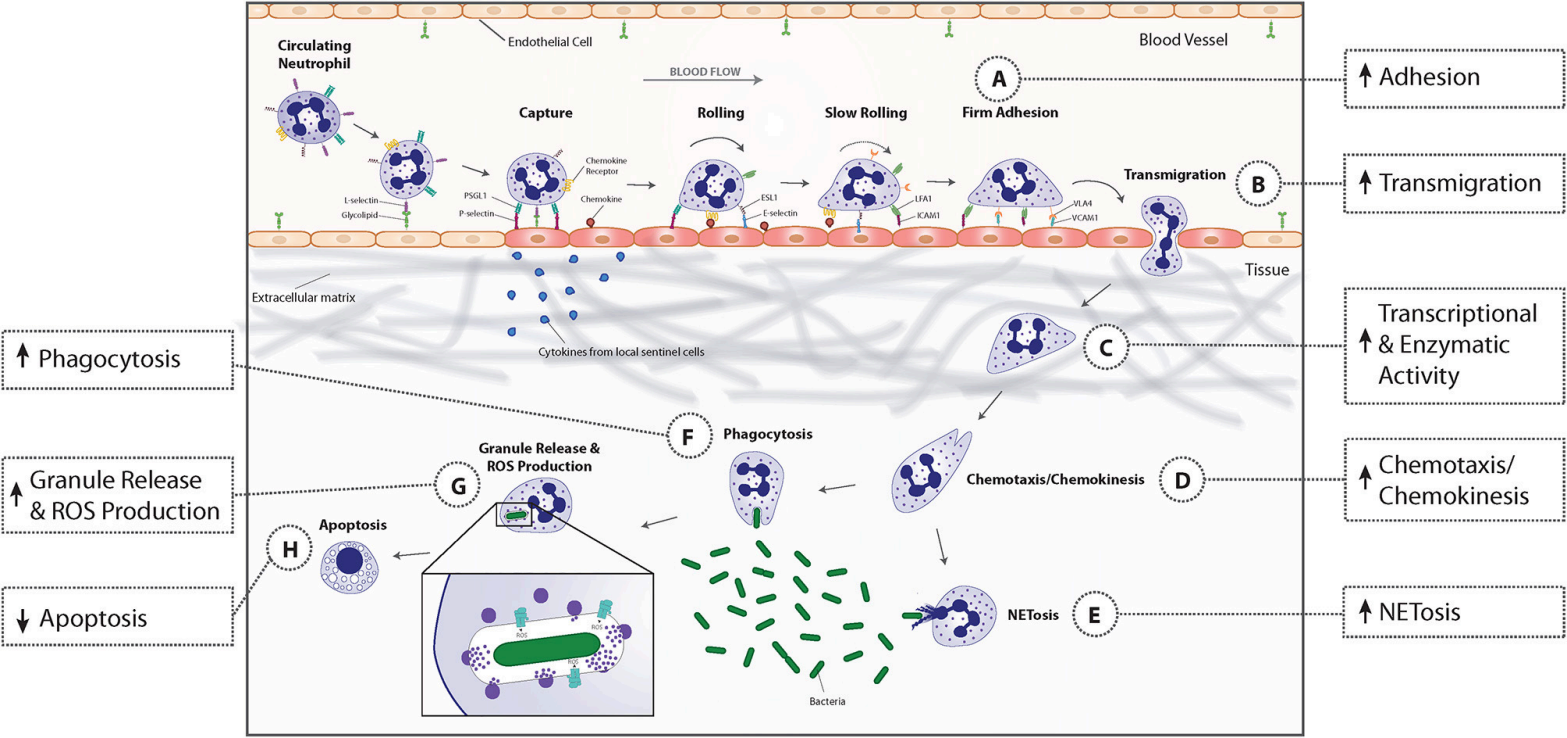
**Priming-associated phenotypic changes and their effect on neutrophil functional responses**. Neutrophils in circulating blood are in a resting state, characterized by a round morphology, non-adherence, minimal transcriptional activity, and a limited capacity to respond to activating stimuli. Microbial entry into tissues or tissue injury induces local immune cells to release pro-inflammatory cytokines that modify endothelial cell adhesion molecule profile and enter the bloodstream to begin priming neutrophils. Upon exposure to these priming agents, neutrophils undergo an increase in enzymatic and transcriptional activity that results in activation and synthesis of inflammatory mediators and enzymes that mediate downstream phenotypic and functional changes. Immediately, neutrophils begin to change their adhesion receptor pattern by shedding selectins, fusing secretory vesicles with the plasma membrane which leads to increased integrin expression, and a rapid increase in the gene expression of several surface receptors that allows newly primed cells to more rapidly adhere to endothelial cells (A). This phenotypic change coupled with the release of granules containing matrix metalloproteases, promotes neutrophil migration into inflamed tissues (B). The priming process continues when neutrophils bind to extracellular matrix proteins (C). Binding of neutrophil extracellular matrix receptors leads to an increase in actin polymerization, available receptors from secretory vesicle degranulation, and intracellular signaling that results in enhanced chemotaxis and chemokinesis (D). When primed neutrophils encounter bacteria, their phagocytic capacity is increased due to the upregulation in the number and affinity of receptors on the plasma membrane (F). By then, ROS production, granule release (G), and NET formation (E) have been primed to augment microbicidal activities. Finally, priming prolongs neutrophil lifespan by activating anti-apoptotic signal transduction pathways and transcription factors that decrease transcription of pro-apoptotic factors (H).

Polymorphonuclear leukocytes, or neutrophils, account for 40–60% of peripheral blood leukocytes in humans (Summers et al., 2010). They play an essential role in the innate immune response, as demonstrated by the development of life-threatening infections or uncontrolled inflammation in individuals with severe neutropenia or genetic disruption of neutrophil anti-microbial capabilities (Kannengiesser et al., 2008; van de Vijver et al., 2012; Moutsopoulos et al., 2014; Nauseef and Borregaard, 2014). Figure 1 shows the multistep process of neutrophil recruitment in response to microbial invasion, including adhesion to vascular endothelium, transmigration into the interstitial space, chemotaxis/chemokinesis toward the site of infection, phagocytosis of pathogens, destruction of microbes within phagosomes by release of antimicrobial granule contents following granule fusion and ROS generation at the phagosomal membrane, and amplification and organization of the inflammatory response. Uncontrolled or prolonged neutrophil activation uses antimicrobial responses to injure normal host cells, leading to pathologic changes to tissues and organs in autoimmune and inflammatory diseases (Nathan, 2006). Consequently, neutrophil activation is normally tightly regulated.

Circulating neutrophils exist in a basal state, characterized by non-adherence, a round morphology, minimal transcriptional activity, and a limited capacity to respond to activating stimuli. That limited response protects against unwarranted inflammatory responses and tissue injury (Sheppard et al., 2005). To effectively clear invading organisms, neutrophils must be capable of mounting rapid, vigorous responses to activating stimuli. The transition to a state of enhanced responsiveness has been termed priming (Condliffe et al., 1998; El-Benna et al., 2008; Wright et al., 2013). It occurs *in vitro* following neutrophil exposure to pro-inflammatory lipids and cytokines, chemokines, mitochondrial contents, and bacterial and viral products (El-Benna et al., 2008). Neutrophil priming *in vitro* represents an *in vivo* phenomena, as primed neutrophils have been identified in humans with infections, rheumatoid arthritis, chronic kidney disease, traumatic injury, and acute respiratory distress syndrome (Bass et al., 1986; McLeish et al., 1996; Ogura et al., 1999; Naegele et al., 2012). Although, substantial circumstantial evidence suggests that primed neutrophils participate in a number of human diseases, direct evidence is lacking. The relative contribution of neutrophil priming to the severity of human inflammatory diseases is an important gap in knowledge that needs to be addressed.

Historically, the term “priming” was primarily used to describe the augmented reactive oxygen species (ROS) generation upon neutrophil stimulation because of the depth of knowledge of molecular mechanisms of NADPH oxidase complex assembly, the ease of measurement of ROS generation, and the importance of ROS to anti-microbial activity. Figure 1 illustrates that primed neutrophils demonstrate a number of phenotypic changes in addition to enhanced NADPH oxidase activation, including granule release, cytokine and lipid synthesis, adhesion and transmigration, enhanced chemotaxis, and delayed apoptosis. Thus, neutrophil priming is not just a transition state in which neutrophils become more responsive to activating stimuli. We believe a new definition of priming is required to include the activation of a subset of neutrophil functions as opposed solely to a heightened state of responsiveness. In this review of the recent advances in neutrophil priming, we will highlight the functional evidence for the activation of a subset of neutrophil functions during priming and review the current state of knowledge of the molecular basis for those phenotypic changes to illustrate this new definition. Our goal is to encourage research that will provide a more complete understanding of priming, leading to identification of new targets for treatment of inflammatory and infectious diseases. Much of our discussion focuses on the effects of TNFα, as studies frequently use that cytokine as a model priming agent. The large number of agents capable of initiating priming of neutrophil respiratory burst activity was recently reviewed (El-Benna et al., 2016). We compare current state of knowledge of the effects of those priming agents on the various phenotypic changes to those induced by TNFα in Table 1.

**Table 1:** **Known priming agents' capacity to induce phenotypic changes in neutrophils**.

**Priming Agent**		**Adhesion**	**Chemotaxis**	**Phagocytosis**	**Granule Release**	**NET formation**	**Apoptosis**	**Inflammatory Mediators**
Chemoattractants	fMLF	↑ (El Azreq et al., 2011)	↑ Halpert et al., 2011	↑ Richardson and Patel, 1995	↑Uriarte et al., 2011	?	No change Klein et al., 2001	↑Browning et al., 1997
	C5a	↑ Jagels et al., 2000	↑ Halpert et al., 2011	↑/↓ Morris et al., 2011; Tsuboi et al., 2011	↑ DiScipio et al., 2006	?	↓ Perianayagam et al., 2002	↑ Finsterbusch et al., 2014
	LTB4	↑ Eun et al., 2011	↑ Afonso et al., 2012	↑ Mancuso et al., 2001	↑ Kannan, 2002	?	↓ Klein et al., 2001	↑ Finsterbusch et al., 2014
	PAF	↑ Kulkarni et al., 2007	↑ Shalit et al., 1987	↑ Rosales and Brown, 1991	↑ Andreasson et al., 2013	?	↓ Khreiss et al., 2004	↑ Aquino et al., 2016
Cytokines	TNF-α	↑ Bouaouina et al., 2004	↑ Montecucco et al., 2008	↑ Della Bianca et al., 1995	↑ McLeish et al., 2013, 2017	↑ Hazeldine et al., 2014	↑/↓ Murray et al., 1997	↑ Bauldry et al., 1991; Jablonska et al., 2002b
	GM-CSF	↑ Yuo et al., 1990	↑ Cheng et al., 2001	↑ Kletter et al., 1989	↑ Kowanko et al., 1991	↑ Yousefi et al., 2009	↓ Klein et al., 2000	↑ DiPersio et al., 1988b; Lindemann et al., 1988
	IFN-γ	↑ Klebanoff et al., 1992	↓ Aas et al., 1996	↑ Melby et al., 1982	↑ Cassatella et al., 1988	?	↑ Perussia et al., 1987	↑ Humphreys et al., 1989
	IL-1β	↑ Brandolini et al., 1997	↑ Brandolini et al., 1997	?	↑ Brandolini et al., 1997	?	?	?
	IL-8	↑ Detmers et al., 1990	↑ Baggiolini and Clark-Lewis, 1992	↑ Richardson and Patel, 1995	↑ Baggiolini and Clark-Lewis, 1992	↑ Hazeldine et al., 2014; Podaza et al., 2016	↓ Acorci et al., 2009	?
	IL-15	?	↑ Mastroianni et al., 2000	↑ Musso et al., 1998	?	?	↓ Mastroianni et al., 2000	↑ Musso et al., 1998; Jablonska et al., 2001
	IL-18	↑ Wyman et al., 2002	?	?	?	?	↑ Wyman et al., 2002	↑ Jablonska et al., 2002a
	IL-33	?	↑ Le et al., 2012	↑ Lan et al., 2016	?	?	?	?
	Adiponectin	?	?	?	?	?	?	?
Microbial Products	LPS	↑ Hayashi et al., 2003; Sabroe et al., 2003	↓/↑ Fan and Malik, 2003; Hayashi et al., 2003	↑ Hayashi et al., 2003	↑ Fittschen et al., 1988; Ward et al., 2000	↑ Hazeldine et al., 2014	↓ Klein et al., 2001	↑ Cassatella, 1995)
	LAMs	?	No change Fietta et al., 2000	No change Fietta et al., 2000	↑ Faldt et al., 2001	?	?	?
	Lipopeptide	↑ Hayashi et al., 2003; Sabroe et al., 2003	↓/↑ Aomatsu et al., 2008	↑ Hayashi et al., 2003	↑/↓ (Whitmore et al., 2016)	?	Minimal effect Sabroe et al., 2003	↑/↓ Whitmore et al., 2016
	Flagellin	↑ Hayashi et al., 2003	↓ Hayashi et al., 2003	↑ Hayashi et al., 2003	?	?	No change/↓ Francois et al., 2005; Salamone et al., 2010	↑/↓ Hayashi et al., 2003
Others	ATP	?	↑ Ding et al., 2016	?	↑ Aziz et al., 1997; Meshki et al., 2004	?	?	?
	Substance P	↑ Dianzani et al., 2003	↑ Marasco et al., 1981; Perianin et al., 1989	?	↑ Marasco et al., 1981	?	↓ Bockmann et al., 2001	↑ Perianin et al., 1989; Wozniak et al., 1989
	CL097, CL075	?	?	?	↑ Makni-Maalej et al., 2012	?	?	?
	Adhesion	**–**	**–**	↑ Kasorn et al., 2009	↑ Xu and Hakansson, 2002	?	↓ Mayadas and Cullere, 2005	↑ Steadman et al., 1996

*Table 1 shows the phenotypic changes induced by agents known to prime neutrophil respiratory burst activity. All the priming agents listed in this table are known inducers of enhanced NADPH oxidase activity. ↑refers to an increase in activity compared to unprimed neutrophils, ↓refers to a decrease in activity, and ? indicates an unknown effect of priming agent*.

## Phenotypic changes during priming

### Respiratory burst activity

For decades, enhanced respiratory burst activity has defined a primed neutrophil. The respiratory burst generates ROS through conversion of molecular oxygen to superoxide by the multi-component NADPH oxidase complex. The oxidase is comprised of three membrane subunits (gp91^phox^/NOX2, p22^phox^, and Rap1A) and four cytosolic proteins (p47^phox^, p67^phox^, p40^phox^, and Rac2). Spatial separation of the membrane and cytosolic components maintains enzymatic inactivity in resting neutrophils. Upon stimulation, the cytosolic components translocate to the membrane to form the catalytically active enzyme complex. Phosphorylation of cytosolic NADPH oxidase components is necessary for translocation of those components to the plasma membrane. One of the major targets of phosphorylation is the p47^phox^ subunit. Phosphorylation of a number of serines (Ser^303^–Ser^379^) early in the activation process facilitates p47^phox^ docking to membrane and cytosolic oxidase components, leading to assembly of the functional oxidase (El-Benna et al., 1994, 1996; Groemping et al., 2003).

Non-receptor tyrosine kinases and p38 mitogen-activated protein kinase (MAPK) are signaling molecules that participate in priming respiratory burst activity by TNFα (El-Benna et al., 1996; McLeish et al., 1998; Forsberg et al., 2001; Dewas et al., 2003; Boussetta et al., 2010). Inhibition of tyrosine kinase activity blocks the activation of p38 MAPK by TNFα (McLeish et al., 1998), indicating that tyrosine kinases participate in priming by activating p38 MAPK. TNFα-mediated activation of the p38 MAPK pathway contributes to priming by enhancing plasma membrane translocation of the cytosolic components of the NADPH oxidase and by increasing expression of the plasma membrane oxidase components. Enhanced translocation of cytosolic components results from p38 MAPK-dependent phosphorylation of Ser^345^ on p47^phox^. Phosphorylation of Ser^345^ initiates a series of conformational changes in p47^phox^ that result in hyperactivation of the NADPH oxidase. The initial event is binding of the prolyl isomerase Pin1 to the phospho-Ser^345^ site (Boussetta et al., 2010). This produces a conformational change in p47^phox^ that exposes additional amino acids for phosphorylation by protein kinase C (PKC). Phosphorylation by PCK produces a second conformational change that promotes p47^phox^ binding to p22^phox^. That interaction leads to translocation and assembly of all the cytosolic oxidase components with the membrane NADPH oxidase components. Pin1 is also involved in priming by GM-CSF and CL097, a TLR8 agonist (Makni-Maalej et al., 2012, 2015). Unlike TNFα, GM-CSF induces phosphorylation of Ser^345^ on p47^phox^ through activation of ERK1/2, not p38 MAPK (Boussetta et al., 2010; Makni-Maalej et al., 2015). This observation indicates that multiple signal transduction pathways induce the same molecular events required for priming. Those redundant signal transduction pathways are unlikely to serve as effective therapeutic targets.

Over a decade ago, it was suggested that TNFα and LPS play a role in respiratory burst priming by influencing membrane trafficking (DeLeo et al., 1998; Ward et al., 2000). Direct confirmation was provided recently by selectively blocking exocytosis prior to priming through the use of cell-permeable, peptide inhibitors of SNARE protein interactions (Uriarte et al., 2011; McLeish et al., 2013). Those studies determined that exocytosis of secretory vesicles and gelatinase granules is required for priming by TNFα and platelet activating factor. Exocytosis could be contributing to priming by increasing plasma membrane expression of receptors, signaling molecules, and/or NADPH oxidase membrane components. The role of receptor and signaling molecule expression in priming was examined by measuring the activation of p38 MAPK and ERK1/2 in neutrophils primed during inhibition of exocytosis (Uriarte et al., 2011). The absence of granule exocytosis had no effect on activation of either MAPK, indicating that increased expression of receptors and signaling molecules does not contribute to priming (Uriarte et al., 2011). Inhibition of Pin1 activity had no effect on neutrophil granule exocytosis (McLeish et al., 2013). We interpret those studies to indicate that enhanced translocation of cytosolic oxidase components and increased expression of membrane oxidase components are independent events, both of which are required for priming.

A second membrane trafficking event that participates in priming respiratory burst activity is clathrin-mediated endocytosis. Moreland and colleagues reported that the NADPH oxidase assembles on endosomes, and the subsequent H_2_O_2_ production was required for neutrophil priming by endotoxin (Moreland et al., 2007; Volk et al., 2011; Lamb et al., 2012). We have confirmed those observations and determined that endocytosis is an upstream event in neutrophil granule exocytosis.

### Neutrophil granule release

Neutrophil granules are divided into four classes based on granule density and contents (Borregaard and Cowland, 1997; Lominadze et al., 2005; Rørvig et al., 2013). Secretory vesicles are created by endocytosis, while gelatinase (tertiary), specific (secondary), and azurophilic (primary) granules are formed from the *trans*-Golgi network during neutrophil maturation (Borregaard, 2010). Granule subsets undergo an ordered release based on stimulus intensity, termed graded exocytosis (Sengelov et al., 1993, 1995). Secretory vesicles undergo exocytosis more easily and completely than gelatinase granules. Specific and azurophilic granules, which contain toxic anti-microbial components, undergo the most limited exocytosis. An *in vivo* study showed that neutrophils migrating into a skin blister created in normal human subjects release nearly 100% of their secretory vesicles, 40% of gelatinase granules, 20% of specific granules, and < 10% of azurophilic granules (Sengelov et al., 1995).

We recently reported that TNFα directly stimulated exocytosis of secretory vesicles and gelatinase granules (McLeish et al., 2017). Those results support previous studies showing that exocytosis of secretory vesicles and gelatinase granule is required for TNFα-induced priming (McLeish et al., 2013). Neither TNFα nor fMLF, alone, stimulated exocytosis of specific and azurophilic granules. However, TNFα primed the release of both granule subsets upon subsequent stimulation by fMLF (McLeish et al., 2017). The ability of TNFα to prime exocytosis of azurophilic granules was also reported by Potera et al. (2016). Thus, differential regulation of exocytosis of the four granule subsets by TNFα primes the two major neutrophil anti-microbial defense mechanisms for enhanced release of ROS and toxic granule contents, while protecting against cell injury from inappropriate release of those toxic products. On the other hand, Ramadass et al. showed that GM-CSF both stimulated and primed exocytosis of gelatinase, specific, and azurophilic granules in mouse neutrophils (Ramadass et al., 2017). The basis for differences between TNFα and GM-CSF could be due to disparate capabilities of priming agents or to species differences.

Proteins that control priming by regulating exocytosis have only recently been identified. As pharmacologic inhibition of p38 MAPK prevents TNF-α stimulated exocytosis (Mocsai et al., 1999; Uriarte et al., 2011; McLeish et al., 2013), we employed a phosphoproteomic analysis by mass spectrometry to identify proteins phosphorylated by the p38 MAPK pathway during TNFα stimulation (McLeish et al., 2017). Four of the proteins identified, Raf1, MARCKS, ABI1, and myosin VI, were previously shown to be involved in exocytosis in various cells. We confirmed that Raf1 participates in TNFα-stimulated exocytosis. Catz and colleagues used neutrophils from transgenic mice to identify Rab27a and its target, Munc13-4, as mediators of neutrophil exocytosis stimulated by GM-CSF (Ramadass et al., 2017). They showed that Rab27a, but not Munc13-4, was required for GM-CSF priming of exocytosis to subsequent stimulation by TLR agonists or formyl peptides. Thus, the mechanisms that control neutrophil exocytosis during priming offer potential targets for intervention in inflammatory processes in which neutrophil priming is involved.

### Adhesion, chemotaxis, and phagocytosis

As shown in Figure 1, microbial invasion or tissue injury releases pathogen-associated molecular pattern (PAMPs) or damage-associated molecular pattern (DAMPs) molecules that induce sentinel immune cells to release pro-inflammatory cytokines. Those cytokines modify both endothelial cell and neutrophil adhesion molecule expression to facilitate the capture of circulating neutrophils and to mediate their migration into tissues. As shown in Table 1, all priming agents for which there are data directly activate neutrophil adhesion. However, differential regulation of adhesion molecule expression and activation by different priming agents may produce different rates of neutrophil adhesion and migration efficiency. For example, neutrophil exposure to TNFα increases plasma membrane expression of the β2 integrin receptor, CD11b/CD18, through exocytosis of secretory vesicles; decreases expression of the selectin receptor CD62-L through receptor shedding; and induces sustained activation of CD11b/CD18 through inside-out signaling (Condliffe et al., 1996; Swain et al., 2002). On the other hand, PAF increases surface expression of the CD11b/CD18, has no effect on selectin expression, and induces only transient activation of CD11b/CD18 (Berends et al., 1997; Khreiss et al., 2004). The *in vivo* significance of those differences in adhesion molecule expression and activation remains to be determined.

With the exception of IFNγ, neutrophil chemotaxis is enhanced by all priming agents for which there are data (Table 1). In addition to increased expression of adhesion molecules and receptors resulting from exocytosis, priming agents increase actin reorganization (Borgquist et al., 2002), and enhances chemokinesis and chemotaxis (Montecucco et al., 2008; Yao et al., 2015). For example, treatment of neutrophils with PAF, IL-8, or TNFα, alone, induces chemokinesis, while subsequent exposure to an fMLF gradient leads to enhanced neutrophil chemotaxis (Drost and MacNee, 2002). Additionally, TNFα-primed neutrophils gain the ability to migrate toward the chemokine CCL3, which is found in inflammatory sites, but is normally not a neutrophil chemo attractant (Montecucco et al., 2008).

Neutrophil adhesion through both the engagement of neutrophil β2 integrin receptors with endothelial cell adhesion molecules and the binding of neutrophil receptors with extracellular matrix proteins primes respiratory burst activity (Stanislawski et al., 1990; Dapino et al., 1993; Liles et al., 1995). Neutrophil adhesion induces other priming phenotypes, including exocytosis of secretory vesicles and gelatinase granules and a reduced rate of apoptosis (Hu et al., 2004; McGettrick et al., 2006; Paulsson et al., 2010). Thus, transmigration of neutrophils into the extravascular space can be expected to directly induce some of the features of priming.

When neutrophils arrive at the site of infection, they demonstrate increased phagocytosis due to upregulation in the number and affinity of phagocytic receptors (Condliffe et al., 1998; Rainard et al., 2000; Le et al., 2012). Table 1 lists the effects of specific priming agents on phagocytosis. Exposure of bovine neutrophils to the combination of two priming agents, TNFα and C5a each at suboptimal concentrations, enhanced both the rate of phagocytosis and the killing capacity toward serum opsonized *Staphylococcus aureus* (Rainard et al., 2000); and incubation of human neutrophils with insulin-like growth factor I (IGF-I) results in a significant increase in phagocytosis of both IgG-opsonized *S. aureus* and serum-opsonized *Candida albicans* (Bjerknes and Aarskog, 1995). Increased neutrophil phagocytosis is dependent on the concentration and incubation time with IGF-1, and is due to increased complement receptor (CR) 1 and CR3 expression. IGF-1 enhances Fcγ receptor-dependent phagocytosis through increased receptor function and activation, while Fcγ receptor expression is unchanged (Bjerknes and Aarskog, 1995). Thus, neutrophil exposure to the complex milieu of priming agents *in vivo* is likely to produce additive or synergistic changes in functional responses. Defining neutrophil responses in that complex environment will require application of systems biology methodologies.

### Neutrophil extracellular trap (NET) formation

Since their first description in 2004, neutrophil extracellular traps (NETs) have received intense investigation. Although, the majority of studies have measured NET formation by resting neutrophils, neutrophils from normal subjects primed by TNFα *in vitro* demonstrated robust NET formation following a 3 h exposure to anti-neutrophil cytoplasmic antibodies (Kessenbrock et al., 2009). Enhanced NET formation in primed neutrophils is supported by other *in vitro* studies using GM-CSF and TNFα (Yousefi et al., 2009; Hazeldine et al., 2014). The effect of priming agents on NET formation is listed in Table 1.

Despite their original classification as the third bacterial killing mechanism, current opinion leans toward NETs being important contributors to autoimmunity and tissue injury, rather than antibacterial activity (Sorensen and Borregaard, 2016). *In vivo*, enhanced NET formation following a systemic change in levels of inflammatory cytokines has been described in cancers, multiple sclerosis, and diabetes (Chechlinska et al., 2010; Naegele et al., 2012; Fadini et al., 2016). Using a chronic myelogenous leukemia mouse model, Demers and colleagues reported that non-malignant neutrophils showed enhanced NET formation, leading to increased coagulation and thrombosis (Demers et al., 2012). Priming of NET formation was reproduced in control mice by sequential administration of granulocyte colony-stimulating factor (G-CSF) and LPS. The authors suggested that priming NET formation by systemic cytokines plays a role in cancer progression. While the current literature indicates that enhanced NET formation is a component of neutrophil priming, the functional consequences of that response remain to be determined.

### Secretion of lipid and cytokine mediators

As summarized in Table 1, primed neutrophils demonstrate increased metabolic and transcriptional activity that leads to synthesis of a number of pro- and anti-inflammatory chemokines, cytokines, and lipids. Although, the ability of neutrophils to synthesize those products is less than that of macrophages, the large number of neutrophils present at sites of inflammation is postulated to influence both innate and adaptive immune responses through release of those inflammatory mediators.

Pro-inflammatory lipid mediators like leukotriene B_4_ (LTB_4_) can be produced *de novo* by the arachidonate 5-lipoxygenase (5-LO) pathway in neutrophils and play important roles in aggregation, degranulation, and chemotaxis (O'Flaherty et al., 1979; Flamand et al., 2000). The production of these lipid mediators occurs through a series of biochemical events that primarily take place in the perinuclear region where membrane phospholipids are first converted to arachidonic acid (AA) by the calcium–dependent enzyme phospholipase A2 (PLA_2_) (Luo et al., 2003; Leslie, 2004). The newly synthesized AA is then converted by 5-LO into leukotriene A_4_(LTA_4_), which is the immediate precursor of LTB_4_. Neutrophil production of LTB4 is responsible for a second wave of neutrophil recruitment during inflammation, a process termed “swarming” (Lammermann et al., 2013). This is one of many examples of amplification loops initiated by neutrophils (Nemeth and Mocsai, 2016).

Direct activation of neutrophils by fMLF does not lead to the detectable release of leukotrienes, but priming with GM-CSF, LPS, or TNFα followed by fMLF stimulation significantly increases LTB_4_ release (see Table 1; DiPersio et al., 1988a; Schatz-Munding and Ullrich, 1992; Palmantier et al., 1994; Seeds et al., 1998; Zarini et al., 2006). All three of these priming agents activate PLA_2_ and increase AA release without increasing intracellular Ca^2+^ (DiPersio et al., 1988b; Schatz-Munding and Ullrich, 1992; Zarini et al., 2006). The elevation in available AA substrate leads to prolonged activation of 5-LO and enhanced production of downstream lipid mediators (Surette et al., 1993, 1998; Doerfler et al., 1994). Once produced, LTB_4_ can exert autocrine effects. It primes neutrophil responses to toll-like-receptor (TLR) agonists, resulting in enhanced cytokine (IL-8, TNFα) secretion (Gaudreault et al., 2012). TLR9 mRNA levels are upregulated upon priming with LTB_4_, but there is no increase in surface expression of TLR2, TLR4, or the co-receptors TLR1 and TLR6 following LTB_4_ exposure (Gaudreault and Gosselin, 2009; Gaudreault et al., 2012). Instead, neutrophil LTB_4_-induced hyper-responsiveness is mediated by the potentiation of TLR-induced intracellular signaling. TAK1 and p38 MAPK, which are essential in TLR-activated cytokine release, are phosphorylated and activated following LTB_4_ interaction with its seven transmembrane-spanning receptor.

PAF is another lipid inflammatory mediator whose production is primed in neutrophils. Both LPS and GM-CSF enhance PAF synthesis in response to activating stimuli (Aglietta et al., 1990; Surette et al., 1998). After priming with GM-CSF, there is increased enzymatic activity of acetyl transferase, the enzyme responsible for the synthesis of PAF (Aglietta et al., 1990). However, the pattern of PAF synthesis after LPS priming is attributed to a biphasic, autocrine response. The early peak in production is due to the direct effect of LPS, while the delayed peak is a result of LPS-induced IL-8 and TNF-α release (Bussolati et al., 1997).

Neutrophils modulate inflammation through the release of stored or newly produced cytokines and chemokines (Cassatella, 1999). Exposure of neutrophils to priming agents leads to an increase in synthesis and release of IL-1α, IL-1β, IL-6, IL-8, TNFα, CXCL1, CXCL2, CCL3 (MIP-1α), CCL4 (MIP-1β) (Roberge et al., 1998; Zallen et al., 1999; Jablonska et al., 2002b; Choi et al., 2008; Wright et al., 2013). The inducible synthesis of the majority of cytokines and chemokines results from increased gene transcription (Marucha et al., 1991; Cassatella et al., 1995; Cassatella, 1996, 1999; Fernandez et al., 1996). TNFα, LPS, and GM-CSF increase intra-nuclear translocation of NF-κB, C/EBP, or CREB transcription factors (Cloutier et al., 2007, 2009; Mayer et al., 2013). LPS induces a biphasic production of IL-8. For the first few hours (2–6 h) of exposure, LPS directly stimulates IL-8 synthesis, but the second wave of sustained IL-8 release (up to 18 h) is due to the endogenous release of TNFα and IL-1β (Cassatella et al., 1993).

### Release of neutrophil extracellular vesicles

Cell-derived vesicles represent a mechanism for cell-cell communication. Exosomes are 50–100 nm vesicles released from multivesicular bodies that are involved in antigen presentation and cell-to-cell transfer of receptors or RNA (Gyorgy et al., 2011). Larger vesicles, called microvesicles or microparticles express tissue factors on their surface that are capable of initiating coagulation. Neutrophils undergoing apoptosis or stimulated by chemotactic agents, opsonic receptors, or TNFα release microparticles. However, the microparticles have varying compositions and functional capabilities, depending on the stimulus (Dalli et al., 2013; Johnson et al., 2014; Lorincz et al., 2015). Microparticles obtained from neutrophils stimulated by chemotactic agents or phorbol esters activate cytokine (IL-6) secretion from endothelial cells and platelets (Mesri and Altieri, 1998; Pluskota et al., 2008). Chemotactic peptide-induced microparticles increase secretion of the anti-inflammatory cytokine transforming growth factor-β and interfere with the maturation of monocyte-derived dendritic cells (Gasser and Schifferli, 2004; Eken et al., 2010). Auto-antibody-stimulated release of neutrophil microparticles was suggested to be involved in the pathogenesis of vasculitis (Hong et al., 2012). Additional activities ascribed to neutrophil microparticles include suppression of bacterial growth, activation of endothelial cell cytokine production, altered cytokine profile of natural killer cells and monocytes, and increased coagulation (Mesri and Altieri, 1998; Timar et al., 2013a,b; Pliyev et al., 2014). An understanding of the stimuli and signal transduction pathways leading to formation and release of neutrophil extracellular vesicles and their roles in inflammation remains to be developed.

### Rate of apoptosis

Table 1 indicates that neutrophil apoptosis is variably affected in response to priming agents. While LPS, GM-CSF, IL-8, and LTB_4_ have been found to extend neutrophil lifespan *in vitro*, PAF, fMLF, and IL-6 show no effect, and TNFα shows a biphasic response where it promotes apoptosis during the first 8 h of exposure, followed by a delayed rate of apoptosis at later times (Klein et al., 2000, 2001; Cowburn et al., 2002; Liu et al., 2005; Wright et al., 2014). Primed neutrophils from patients with multiple sclerosis, ANCA-associated vasculitis, and liver cirrhosis show increased apoptosis (Harper et al., 2001; Klimchenko et al., 2011; Naegele et al., 2012), while neutrophils from patients at risk of multiple-organ failure and individuals presenting with septic peritonitis, severe trauma, or septic trauma show a decrease in apoptosis (Ertel et al., 1998; Biffl et al., 1999, 2001; Nolan et al., 2000; Feterowski et al., 2001). Those conflicting reports of the effect of inflammation on apoptosis *in vivo* are likely due to different priming agents involved in different diseases, different responses during the time course of disease, and differences in the neutrophil micro-environment, such as cell density (Hannah et al., 1998).

The mechanisms underlying the effects of priming on neutrophil apoptosis have been partially characterized. As for TNFα, increased rates of apoptosis during the first hours of exposure are associated with activation of caspase cascades (Murray et al., 1997). TNFα also induces an early, PI-3K-mediated increase in mRNA levels for Bad, a member of the BCL2 family that regulates apoptosis. On the other hand, decreased neutrophil apoptosis observed at later time points is associated with a reduction in Bad mRNA levels (Cowburn et al., 2002). GM-CSF, IL-8, LPS, and LTB_4_ decrease the rate of neutrophil apoptosis through activation of ERK1/2 and/or PI-3K/Akt pathways (Klein et al., 2000, 2001). Incubation of neutrophils with fMLF had no effect on the rate of apoptosis, despite activation of both ERK1/2 and Akt (Klein et al., 2001). GM-CSF was also shown to decrease mRNA levels of Bad, while increasing its phosphorylation (Cowburn et al., 2002). A RNA seq study comparing TNFα and GM-CSF priming pathways showed that out of 580 genes differentially expressed between both agents, 58 were implicated in the delay of apoptosis. Thus, each priming agent produced a distinct profile of pro- and anti-apoptotic genes (Wright et al., 2013). The varying rates of neutrophil apoptosis may serve different functions in the inflammatory response. For example, a reduced rate of apoptosis early in the recruitment of neutrophils results in a brisk accumulation of primed neutrophils. On the other hand, an enhanced rate of apoptosis at later time points promotes resolution through loss of active neutrophils and a change in phenotype of monocytes engulfing apoptotic neutrophils.

## Conclusions

The altered neutrophil functions described in this review indicate that priming is a complex phenomenon. Priming involves enhanced respiratory burst, exocytosis, NET formation, and chemotaxis in response to a second stimulus. Priming, however, is not just preparation for an enhanced response to a second stimulus. Priming involves activation of a subset of neutrophil responses, including adhesion, transcription, cytoskeletal reorganization, translocation and expression of receptors, and other molecules, the rate of constitutive apoptosis, metabolic activity, and phagocytosis. The altered neutrophil responses associated with priming primarily result in amplification of the inflammatory response. Although, recruitment of primed neutrophils improves the clearance of invading microbes, the risk of directly injuring surrounding cells is increased. Moreover, the increased synthesis and release of cytokines and lipids by primed neutrophils, combined with increased neutrophil recruitment and life-span, result in an increased local concentration of pro-inflammatory agents. Those agents recruit and prime additional neutrophils, leading to an enhanced innate immune response. Neutrophil-dependent recruitment and activation of dendritic cells and various lymphocyte subsets also enhances the adaptive immune response.

We propose that the current definition of priming, which focuses on a transition state to an enhanced responsiveness to a second stimulus, is too restrictive. Neutrophil priming also results in activation of a subset of neutrophil responses that regulate innate and adaptive immunity. Additionally, neutrophil responses to priming agents vary depending on concentration of the priming agent, time of exposure, and the specific priming agent (Potera et al., 2016; McLeish et al., 2017). It seems likely that neutrophils are exposed to graded concentrations of priming agents as they progress through the multistep process of recruitment, as occurs with chemoattactants. This leads to the hypothesis that, similar to graded granule exocytosis, priming occurs in a graded manner during a neutrophil's journey to the site of inflammation. This graded response allows neutrophils to acquire functions in an ordered manner, as required during recruitment. A fully primed neutrophil that releases a maximal amount of toxic chemicals would occur when an optimal concentration of a priming stimulus is encountered. Combining knowledge of the molecular events with an understanding of priming at a systems level will identify therapeutic targets for neutrophil functions that exacerbate individual diseases, while preserving the functions that participation in host defense.

## Author contributions

Contributed to the writing and editing of the manuscript IM, KM, SU; designed and illustrated the figure IM.

### Conflict of interest statement

The authors declare that the research was conducted in the absence of any commercial or financial relationships that could be construed as a potential conflict of interest.
